# Beach grass to Brassicas: A novel salt-tolerant endophyte finds new roots

**DOI:** 10.1093/plphys/kiaf672

**Published:** 2025-12-22

**Authors:** James M Bradley

**Affiliations:** Assistant Features Editor, Plant Physiology, American Society of Plant Biologists, Rockville, USA; Department of Cell & Systems Biology, University of Toronto, Toronto, ON, M5S 3B2, Canada

During domestication, crops were selected for high yield under favorable soil conditions (Shannon 1997). Today, however, persistent irrigation has rendered an estimated 10% of agricultural land highly saline (Shannon 1997). Continued production on contaminated land typically requires switching to salt-tolerant crops or introgressing genes from salt-tolerant wild relatives, yet this can be labor intensive and often fails to fully recover yield (Shannon 1997). Thus, interest has grown in complementary strategies such as using plant growth–promoting (PGP) microbes as “bioinoculants” that boost plant growth and promote tolerance against abiotic stress (Mukhtar et al. 2019).

In the field, plants exist in association with a regulated microbial community, called the phytomicrobiome (Backer et al. 2018). PGP microbes isolated from the phytomicrobiome are routinely assembled into synthetic microbial communities, or “SynComs,” to inoculate crops for improved plant performance and stress resistance (Finkel et al. 2017). While the mechanistic basis of plant growth promotion is often lacking, some traits are repeatedly associated with PGP microbes (Finkel et al. 2017). These include the modulation of host phytohormones (Orozco-Mosqueda et al. 2020), mineral solubilization through the production of organic acids (Rodríguez and Fraga 1999), and/or reactive oxygen species (ROS) detoxification (Liu et al. 2021). To date, the emphasis has been on isolation of microbes from the phytomicrobiomes of crops, which may miss potential beneficial partnerships. To address this gap, researchers are searching the phytomicrobiome of plants adapted to extreme environments (extremophiles) for novel microbes that could be translated into agricultural settings (Mukhtar et al. 2019). However, to build SynComs from extremophilic PGP microbes that are compatible with crops, it is first necessary to characterize their PGP traits and evaluate whether they can establish effective, beneficial associations with target crop species.

Recently in *Plant Physiology*, Peng et al. (2025) investigated the salt-tolerant plant *Carex pumila* for PGP microbes that might confer tolerance to high salinity when transferred to non–salt-adapted species. Specifically, they looked for endophytic bacteria living within the leaves of *C. pumila*. The authors surface-sterilized the leaves, blended them, spotted the homogenate onto agar media, then subcultured selected bacterial isolates with up to 10% NaCl to identify salt-tolerant isolates (Fig. 1a). One highly salt-tolerant isolate, named JBR1, was sequenced and identified as a novel *Pseudomonas sp*. that formed a distinct clade according to whole-genome phylogenetic analysis.

**Figure 1.**
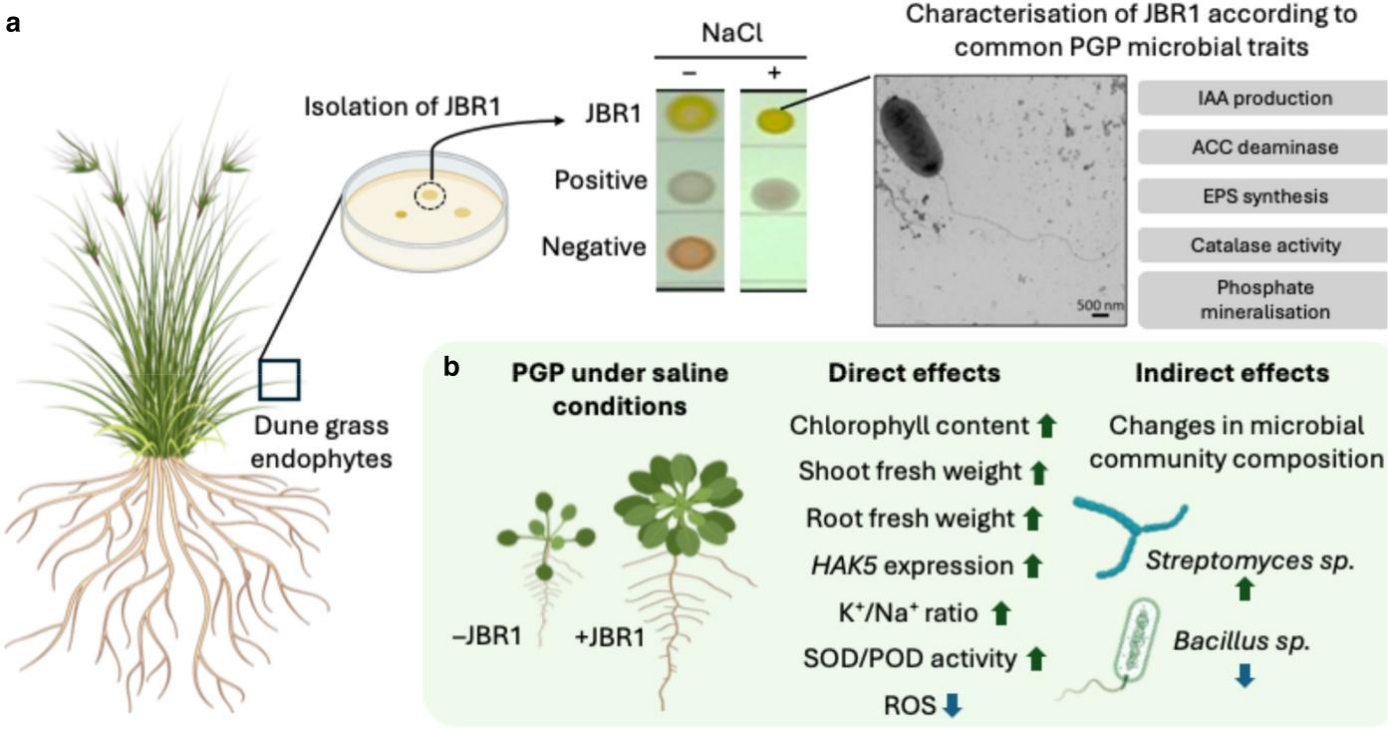
**a)** a novel *Pseudomonas* species (designated JBR1) was isolated on high-salt media from the leaves of the dune grass *Carex pumila*. Image inserts show JBR1 spotted onto media with or without NaCl supplementation, alongside control strains that are either highly resistant (positive) or susceptible (negative) to salt. Enlarged image shows a transmission electron micrograph of a JBR1 cell. Images from Peng et al. (2025). Boxes indicate PGP traits assessed in axenically grown JBR1. **b)** Summary of the direct effects of JBR1 on host plant physiology and indirect effects on microbial community composition. Arrows indicate JBR1 had either a positive (green upwards arrow) or negative (blue downwards arrow) effect. Designed with aid of Biorender.

The authors then investigated traits in JBR1 associated with PGP microbes (Fig. 1a). They found that JBR1 produced the plant hormone auxin (IAA) under saline and non-saline conditions and had ACC (1-aminocyclopropane-1-carboxylic acid) deaminase activity, which increased with increasing NaCl concentrations. ACC deaminase breaks down the precursor of the plant hormone ethylene, which acts as an inhibitor of root growth (Orozco-Mosqueda et al. 2020). Thus, by producing IAA and reducing ethylene levels via ACC deaminase, JBR1 could potentially maintain plant growth under salt stress. Another common trait that can protect plants against salt stress is the production of exopolysaccharides; complex polymer matrices secreted into the soil by bacteria that alter soil properties and nutrient availabilities (Upadhyay et al. 2011). In the context of saline soils, these matrices have the potential to bind cations, including Na^+^, thus alleviating salt stress (Upadhyay et al. 2011). Peng et al. found JBR1 exopolysaccharide production positively correlated with NaCl concentration and remained stable up to 500 mM NaCl, suggesting the potential to protect plants from high Na^+^. Thus, axenically grown JBR1 displayed a range of commonly found PGP traits.

To test whether JBR1 promoted plant growth, the authors inoculated *Arabidopsis thaliana* (Arabidopsis). After 2 weeks, shoot and root fresh weight significantly increased, confirming JBR1 as a PGP microbe under both saline and non-saline conditions (Fig. 1b). Notably, chlorophyll content increased significantly in JBR1-inoculated plants under saline conditions but not under non-saline conditions, suggesting certain JBR1-induced benefits are conferred specifically under salt stress. Peng et al. also visualized and quantified ROS levels in Arabidopsis roots treated with or without JBR1 and found less ROS in the presence of the bacteria. Salt stress can lead to increased ROS production and cellular damage, and organisms often deal with this by upregulating genes encoding enzymes required to detoxify ROS (Liu et al. 2021). Accordingly, the authors detected increased levels of the ROS quenching enzymes, peroxidase and super oxide dismutase, in Arabidopsis shoots (Fig. 1b).

Since JBR1 was able to boost growth under salt stress, Peng et al. investigated ion homeostasis in JBR1-incoulated plants. JBR1 inoculation increased the K^+^/Na^+^ ratio specifically under salt stress, which the authors suggested could help mitigate the high Na^+^ levels. Reasoning that the increase in K^+^ might be due to altered expression of K^+^ transporters, the authors measured transcript levels for the high-affinity K^+^ transporter, *HAK5*. *HAK5* was 1.4-fold induced in JBR1-treated plants under 100 mM NaCl, while only a very slight induction was seen in the absence of salt stress, suggesting a possible mechanism for the increased K^+^ levels. Analogous to *A. thaliana*, the authors then showed the PGP effects of JBR1 extended to the related crop plant *Brassica rapa*. For instance, JBR1 increased shoot and root biomass, as well as improved photosynthetic capacity in *B. rapa*.

Lastly, Peng et al. investigated the potential implications on the wider microbial community. 16S rRNA sequencing in JBR1-inoculated plants grown under saline or non-saline conditions revealed that JBR1 enriched for certain taxa, such as *Streptomyces sp.*, while others such as *Bacillus sp*. were reduced in abundance (Fig. 1b). Indeed, some *Streptomyces* species previously implicated in promoting plant stress resistance, such as *S. griseorubiginosus* (Wang et al. 2021), were among the most highly enriched *Streptomyces* species in JBR1-treated roots under saline conditions. Since *Streptomyces* species have been associated with tolerance to salinity, their recruitment by JBR1 could synergistically boost plant growth under salt stress. Taken together, this suggested a model in which JBR1 promotes plant growth both directly through its effect on plant physiology and indirectly, through its impact on the rhizosphere microbial community (Fig. 1b).

In sum, Peng et al. identified a novel *Pseudomonas* endophyte from the leaves of the salt-tolerant plant *C. pumila*. How JBR1 interacts with plants in the field and within complex synthetic communities will be interesting future questions. More generally, this study reinforces the potential of extremophile plant species as a source of novel PGP microbes. Deploying SynComs in agriculture has the potential to underpin a second green revolution (Backer et al. 2018), but perhaps the full potential of SynComs will only be fully realized when PGP microbes from extremophiles are incorporated.

## Recent related articles in *Plant Physiology*:

Kundu et al (2022) found the plant growth–promoting fungus *Piriformospora indica* induces the production of a plant secondary metabolite, putrescine, to promote growth in tomato. This represents a novel plant growth–promoting trait that does not fit the standard predefined criteria of plant growth–promoting traits as outlined in Fig. 1a.

Yang et al. (2025) found a rhizospheric bacteria isolated from cucumber, *Bacillus amyloliquefaciens*, can promote growth in white lupins through an auxin-dependent promotion of cluster roots under low phosphate conditions.

## Data Availability

There are no new data associated with this article.
